# An unusual cause of infant’s stridor – congenital laryngocele

**DOI:** 10.1186/s40463-020-00430-9

**Published:** 2020-06-01

**Authors:** Ambrus Andrea, Sztanó Balázs, Szabó Miklós, Vasas Béla, Sziller István, Rovó László

**Affiliations:** 1 Department of Otorhinolaryngology, Head and Neck Surgery, University of Szeged, Szeged, Hungary; 2 1st Department of Paediatrics, Semmelweis University, Budapest, Hungary; 3 Department of Pathology, Budapest, Hungary; 4 Szent Imre University Teaching Hospital, Budapest, Hungary

**Keywords:** Congenital stridor, Laryngocele, Airway obstruction

## Abstract

Congenital laryngocele is an uncommon cause of neonatal stridor. There are only a few cases reported in the literature. The authors present a successfully treated case of an infant, whose life could only be saved by urgent tracheostomy. On the 5th postoperative day endoscopic excision and marsupialization provided patent airway. The patient could be decannulated. During follow-up no recurrence was observed.

## Introduction

The neonatal laryngocele is an extremely rare phenomenon, which is defined as an air-filled cystic dilatation of the laryngeal saccule. The incidence is estimated to be 1 per 2.5 million people per year [1]. Although it is a benign lesion, it may cause stridor, respiratory distress and serious airway obstruction in the narrow airway of a newborn which necessitates urgent intervention [2]. The authors present a newborn baby successfully endoscopically treated with congenital laryngocele.

## Case report

**Fig. 1:**
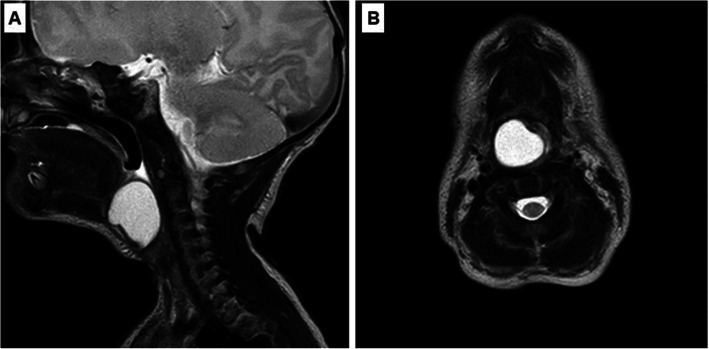
Preoperative MRI. A: Sagittal contrast MRI image shows well-circumscribed, thin-walled fluid attenuation mass. B: Axial contrast MRI image reveals the cystic mass at the level of the glottis

A 5-day-old male newborn was referred to our tertiary department. He was a normal-appearing, 3135 g, full-term baby with no significant prenatal medical history. In another hospital, after birth (Apgar score: 7/7/9), immediately severe stridor and respiratory distress occurred. He required DuoPAP ventilation. Direct laryngoscopy revealed a large cystic mass bulging from the right aryepiglottic fold, which obstructed the glottis. He could not be intubated, so an urgent tracheotomy was performed. MRI described a well-circumscribed, thin-walled fluid attenuation mass, measuring 20 × 18 mm, localized at the level of the glottis (Fig. 1 a, b). No other congenital malformation occurred.

**Fig. 2:**
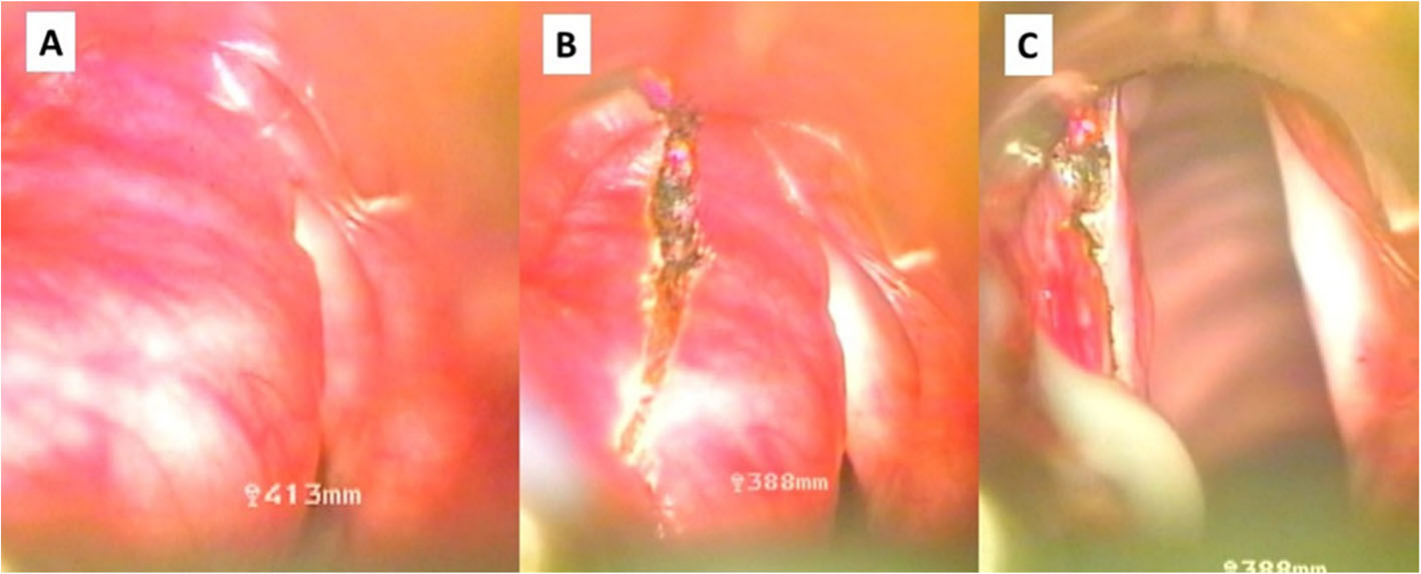
Endoscopic surgery for congenital laryngocele - intraoperative photos. A: Cystic mass of the left false vocal cord cause severe obstruction. B: CO2 laser excision. C: After thick mucoid fluid came out, and marsupialization was done, the asymmetry of the supraglottis disappeared

**Fig. 3:**
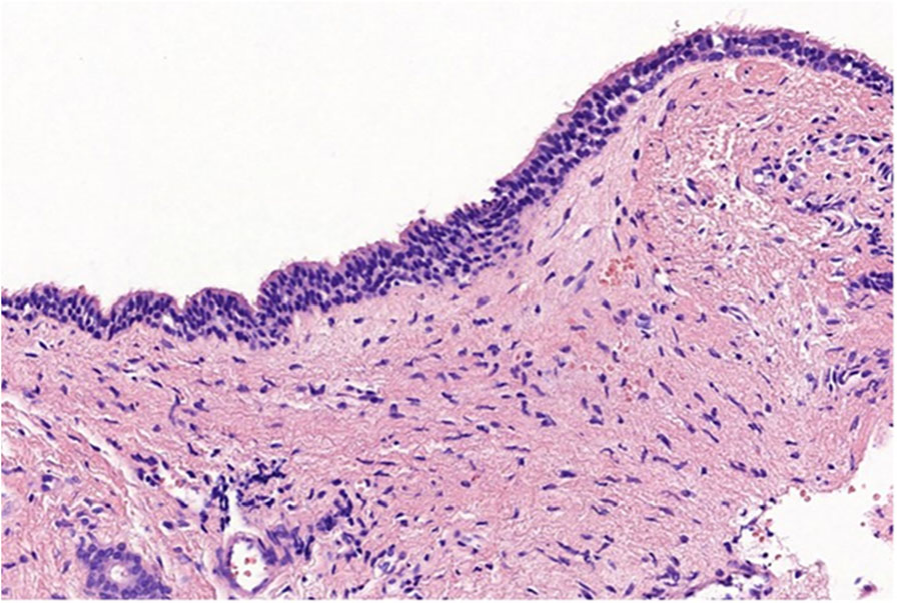
Histologic finding. A hematoxylin-eosin (H&E) stained slide demonstrates the cyst lining, which is comprised of a ciliated pseudostratified columnar epithelium without atypia. There was no mucin accumulation observed in the lumen

After his admission a direct laryngoscopy under general anaesthesia was performed: a swollen cystic mass bulging from the right pharyngoepiglottic and aryepiglottic folds was found (Fig. 2 a). The cyst was incised by ultra-pulse mode CO2 laser (Dhaesin U-40, Dhaesin Enterprise, South Korea; 315 W, 90 μs, 20 ms) (Fig. 2b). Thick mucoid fluid came out, then marsupialization was done. The supraglottic mass which caused laryngeal asymmetry immediately disappeared (Fig. 2c). At the end of the surgery, the baby was decannulated, but he was intubated for four days with a 3.5 cuffed tracheal tube. Parenteral antibiotic (Clindamycin 5 mg/ kg /3 times a day, Cefotaxime 25 mg/ kg/ 2 times a day) was administered for 4 days. After his extubation no dyspnea re-occurred and his stoma closed spontaneously. Postoperative period was uneventful. Control endoscopy performed on the 7th postoperative day revealed no recurrence. During the 6-month follow up period the growing of the baby was appropriate and his parents noticed no voice disturbance. Histologic report confirmed the diagnosis of a congenital laryngocele. (Fig. 3).

## Discussion

The first air-containing ‘tumors’ of the neck were reported in 1829. The first laryngeal cyst in a newborn was published in 1876 [3, 4]. The first reports were based on postmortem diagnosis - all newborns died with any type of airway obstruction. Since 1970s with modern neonatology, the introduction of intubation and advanced endoscopic diagnostic and microsurgical techniques the patients’ life could have been saved.

Laryngocele is an abnormal dilatation of the appendix of the laryngeal ventricle and is classified as internal and mixed or combined type according to its relationship with the thyrohyoid membrane. In reviewing the English-language literature and focusing on the pediatric population, there are very few cases of successfully treated neonatal laryngoceles. Embryologically, the ventricle of the larynx and the saccule develops at the end of the second intrauterine month as a secondary outpouching from the laryngeal lumen. Congenitally large saccule, weakness of the periventricular connective tissues, and the thyroepiglottic and aryepiglottic muscles are accepted as predisposing factors [2].

Airway obstruction cannot always be diagnosed or predicted prenatally [2]. The clinical features of this rare pathology depend on the size and type of laryngocele and are highly variable. In mild cases it is usually asymptomatic. But it may cause severe respiratory distress and inspiratory stridor and become a life-threatening event due to mechanical obstruction of the narrow and small airway of a neonate [5, 6].

The diagnosis of laryngocele mainly depends on physical examination, symptoms and laryngeal examination by direct laryngoscopy [2]. High resolution imaging modalities such as computed tomography or magnetic resonance imaging may help to delineate the structures involved and the exact location of the mass [7].

If the patient has respiratory distress and dyspnea, the airways should immediately be secured by intubation or tracheostomy. In our case the infant could not be intubated; only the urgent tracheostomy could save his life. Various surgical techniques have been utilized such as external approach, endolaryngeal microlaryngoscopy, carbon-dioxide laser excision and transoral robotic surgery [1, 6–8]. Transoral laser endoscopic excision and marsupialization of the cyst’s wall is a minimally invasive solution. Temporary intubation and administration of antibiotics prevented postoperative edema. Although Myssiorek et al. reported that marsupialization has a higher risk of recurrence, during the 6 month follow-up there was no evidence of laryngocele persistence [3, 9, 10].

## Conclusion

Congenital laryngocele is a rare cause of laryngeal dyspnea in neonates. In severe cases urgent surgical intervention must be performed because of the life-threatening airway obstruction. Laryngoscopy may establish the proper diagnosis. Laser excision with complete removal of the cyst’s wall under jet ventilation anesthesia might be a successful treatment without performing a tracheostomy.

## Data Availability

Video documentation of the pre- and postoperative status was achieved.
