# Correction to: An unusual cause of infant’s stridor – congenital laryngocele

**DOI:** 10.1186/s40463-020-00441-6

**Published:** 2020-06-25

**Authors:** Andrea Ambrus, Balázs Sztanó, Miklós Szabó, Béla Vasas, István Sziller, László Rovó

**Affiliations:** 1 Department of Otorhinolaryngology, Head and Neck Surgery, University of Szeged, Szeged, Hungary; 2 1st Department of Paediatrics, Semmelweis University, Budapest, Hungary; 3 Department of Pathology, University of Szeged, Szeged, Hungary; 4 Szent Imre University Teaching Hospital, Budapest, Hungary

**Correction to: J Otolaryngol Head Neck Surg (2020) 49:34**

**https://doi.org/10.1186/s40463-020-00430-9**

Following publication of the original article [1], the authors identified an error in the author group. The given name and family name of all authors were erroneously transposed.

The incorrect author names are: Ambrus Andrea, Sztanó Balázs, Szabó Miklós, Vasas Béla, Sziller István and Rovó László

The correct author names are: Andrea Ambrus, Balázs Sztanó, Miklós Szabó, Béla Vasas, István Sziller and László Rovó

The author group has been updated above and the original article [1] has been corrected.
